# Notes and Notices

**Published:** 1884-02

**Authors:** 


### NOTES AND NOTICES.

THE NEW YORK HOMŒOPATHIC MEDICAL SOCIETY.—The Thirty-third Annual Meeting of this Society will be held at Albany, on the 12th and 13th of this month. Annual address by the President, Everett Hasbrouck, M. D.

A. P. HOLLETT, *Secretary.*

THE REPERTORY OF CHARACTERISTICS will be ready for mailing by March 1st. It gives the characteristic conditions of aggravation and amelioration, and is based on Bœnninghausen's celebrated " Pocket-book." Will be mailed free to all subscribers who have paid their subscriptions.

THE COUGH REPERTORY.—With this issue we give the title-page, list of remedies, etc., of the cough repertory, thereby completing the work proper. A supplement of some thirty pages is to follow, which gathers up all stray matter omitted from the repertory proper.

